# Natural frequency tree- versus conditional probability formula-based training for medical students’ estimation of screening test predictive values: a randomized controlled trial

**DOI:** 10.1186/s12909-024-06209-0

**Published:** 2024-10-24

**Authors:** Soela Kim, Soyun Kim, Yong-jun Choi, Young Kyung Do

**Affiliations:** 1 Institute of Health Policy and Management, Seoul National University Medical Research Center, Seoul, South Korea; 2 Department of Social and Preventive Medicine, Hallym University College of Medicine, Chuncheon, South Korea; 3 Institute of Social Medicine, Hallym University College of Medicine, Chuncheon, South Korea; 4 Department of Health Policy and Management, Seoul National University College of Medicine, Seoul, South Korea

**Keywords:** Probabilistic reasoning, Predictive value of screening tests, Natural frequency, Conditional probability, Medical students

## Abstract

**Background:**

Medical students and professionals often struggle to understand medical test results, which can lead to poor medical decisions. Natural frequency tree-based training (NF-TT) has been suggested to help people correctly estimate the predictive value of medical tests. We aimed to compare the effectiveness of NF-TT with conventional conditional probability formula-based training (CP-FT) and investigate student variables that may influence NF-TT’s effectiveness.

**Methods:**

We conducted a parallel group randomized controlled trial of NF-TT vs. CP-FT in two medical schools in South Korea (a 1:1 allocation ratio). Participants were randomly assigned to watch either NF-TT or CP-FT video at individual computer stations. NF-TT video showed how to translate relevant probabilistic information into natural frequencies using a tree structure to estimate the predictive values of screening tests. CP-FT video showed how to plug the same information into a mathematical formula to calculate predictive values. Both videos were 15 min long. The primary outcome was the accuracy in estimating the predictive value of screening tests assessed using multiple-choice questions at baseline, post-intervention (i.e., immediately after training), and one-month follow-up. The secondary outcome was the accuracy of conditional probabilistic reasoning in non-medical contexts, also assessed using multiple-choice questions, but only at follow-up as a measure of transfer of learning. 231 medical students completed their participation.

**Results:**

Overall, NF-TT was not more effective than CP-FT in improving the predictive value estimation accuracy at post-intervention (NF-TT: 87.13%, CP-FT: 86.03%, *p* = .86) and follow-up (NF-TT: 72.39%, CP-FT: 68.10%, *p* = .40) and facilitating transfer of training (NF-TT: 75.54%, CP-FT: 71.43%, *p =* .41). However, for participants without relevant prior training, NF-TT was more effective than CP-FT in improving estimation accuracy at follow-up (NF-TT: 74.86%, CP-FT: 58.71%, *p =* .02) and facilitating transfer of learning (NF-TT: 82.86%, CP-FT: 66.13%, *p =* .04).

**Conclusions:**

Introducing NF-TT early in the medical school curriculum, before students are exposed to a pervasive conditional probability formula-based approach, would offer the greatest benefit.

**Trial registration:**

Korea Disease Control and Prevention Agency Clinical Research Information Service KCT0004246 (the date of first trial registration: 27/08/2019). The full trial protocol can be accessed at https://cris.nih.go.kr/cris/search/detailSearch.do?seq=15616&search_page=L.

**Supplementary Information:**

The online version contains supplementary material available at 10.1186/s12909-024-06209-0.

## Background

Medical professionals have been reported to have an inadequate ability to draw statistical inferences from medical test results. In previous studies, they gave various incorrect answers when asked about positive predictive values of medical tests—the conditional probability of having a disease when a test result is positive [1–14]. In fact, estimating predictive values involves Bayesian reasoning and requires competency in understanding and combining statistical information on disease prevalence in the population tested (i.e., a prior probability of disease) and test performance characteristics (e.g., sensitivity and specificity), which are also conditional probabilities [15–17]. Unfortunately, medical students, as well as professionals, often confuse positive predictive value with sensitivity [5] and also do not consider the disease prevalence [11, 18]. These errors together create conditions conducive to overestimating positive predictive values [1, 4, 13, 19]. 

Predictive value estimation errors can have serious consequences for not only clinical reasoning but also patient counseling and public health policies. For example, physicians’ overestimation of the positive predictive values of screening tests, combined with their insufficient explanation of the probabilistic nature of test results, might cause tested people considerable emotional distress and the costs associated with seeking further medical care that would have been unnecessary. Such negative consequences could be more pronounced in cases of uncommon, socially stigmatized diseases like HIV and cancer [6, 20, 21]. The recent COVID-19 pandemic also reminded us of the importance of clearly understanding the predictive values of medical screening tests for disease prevention and control [22–25]. In populations where the prevalence of COVID-19 and the positive predictive value of COVID-19 screening are relatively low, implementing a mass testing policy without carefully considering the possibility of false-positive results can lead to many non-infected individuals unnecessarily self-isolating and missing out on critical on-site learning and work [26]. Furthermore, recommending and distributing at-home COVID-19 tests without any measures to assist in accurately interpreting test results may eventually undermine pandemic-control efforts by allowing people to be falsely reassured by a negative test result while ignoring the reason for being tested (i.e., a high prior probability of infection in the population) [27]. Given the significance and scope of the problems that inaccurate estimations of predictive values may cause, it is vital to better equip students and professionals in the healthcare field with the ability to interpret medical test results based on known probabilistic information.

Medical training may contribute, at least in part, to the errors in estimating positive predictive value. In the current medical school curricula, a mathematical formula based on Bayes’ theorem is widely used in training, and students mainly learn how to plug all relevant probabilistic information directly into the formula to calculate predictive values [28]. However, decision scientists have challenged this formula-based approach, instead suggesting training with natural frequencies. Natural frequency tree-based training (NF-TT) is one such approach. NF-TT teaches students how to use tree diagrams to visually represent Bayesian reasoning tasks, which entails converting probabilistic information into natural frequencies [2, 29–31]. A rich body of literature on Bayesian reasoning, including studies on presentation formats and training methods across various populations, demonstrates that both the use of natural frequencies and tree diagram visualization can independently improve Bayesian reasoning [2, 6, 7, 14, 16, 31–41]. Thus, NF-TT, which combines the use of natural frequencies with tree diagram visualization, can offer a synergistic effect of these two elements. Importantly, tree diagrams are not the only visual tool facilitates Bayesian reasoning. Other tools, such as icon arrays and frequency grids, can also effectively convert probabilities into natural frequencies [16, 42]. However, tree diagrams offer a distinct practical advantage in their simplicity and ease of creation by hand with paper and pencil, making them particularly suitable for real-world applications. Several empirical investigations were conducted during the early 2000s involving diverse populations, including medical students and gynecologists in Germany [2, 29, 30, 43]. These studies collectively established that NF-TT was more effective than conventional conditional probability formula-based training (CP-FT).

Our study builds upon the existing body of research in several ways. First, we examine the effectiveness of NF-TT in South Korean medical education, where how to estimate predictive values of medical tests is typically taught using mathematical formulas, although 2 × 2 frequency tables are often used to conceptually demonstrate diagnostic accuracy measures (i.e., specificity, sensitivity, and predictive values). This educational context provides an opportunity to examine how NF-TT might complement or improve upon the current prevalent practice (represented by CP-FT). Second, while NF-TT’s superiority over CP-FT is well-established, its effectiveness shows notable variation across studies [2, 29, 30, 43]. To better understand this variability, we investigate how prior training moderates the effectiveness of NF-TT. Specifically, by focusing on this easily observable student segmentation variable, we aim to provide practical guidance for medical educators in optimizing NF-TT implementation. Additionally, following the approach of a previous study [30], we examine how these skills acquired through training persist over time and transfer to Bayesian reasoning in non-medical contexts.

## Methods

### Study setting and eligibility criteria

We conducted a parallel randomized controlled trial with a one-month follow-up (a 1:1 allocation ratio) to compare NF-TT with CP-FT. The specific sites of this study were two medical schools located in Seoul and Chuncheon, South Korea, respectively, which will be referred to as School A and School B hereafter. Like most South Korean medical schools, both schools offer six-year programs that students enter after graduating from high school and four-year graduate-entry programs for students with a bachelor’s degree in a field other than medicine. The curriculum of the six-year programs consists of two years of a pre-medical course and four years of a medical course, whereas that of the graduate-entry programs does not include the pre-medical component. Similar to other South Korean medical schools, when teaching Bayesian reasoning in clinical medicine, both schools use the traditional formula-based training approach. However, they differ in when the training takes place in their respective curriculum. School A offers a class where students learn about the common indices of test accuracy (i.e., sensitivity, specificity, false-positive/negative, and positive/negative predictive values) in the first semester of the first year of the medical course (right after completing two years of pre-medical course); School B offers the class in the first semester of the second and the last years of the medical course.

Students enrolled in either School A or B were eligible for study participation if they agreed to provide contact details (e.g., email address and phone number) to receive study invitations and bank details (e.g., bank account number, bank name) to receive monetary compensation for study participants. Exclusion criteria included being less than 19 years old and not agreeing with all aspects of the study protocol and methods. We used a quota sampling strategy based on year in school to ensure equal representation of students from all six years in medical school.

### Procedure and intervention

**Fig. 1:**
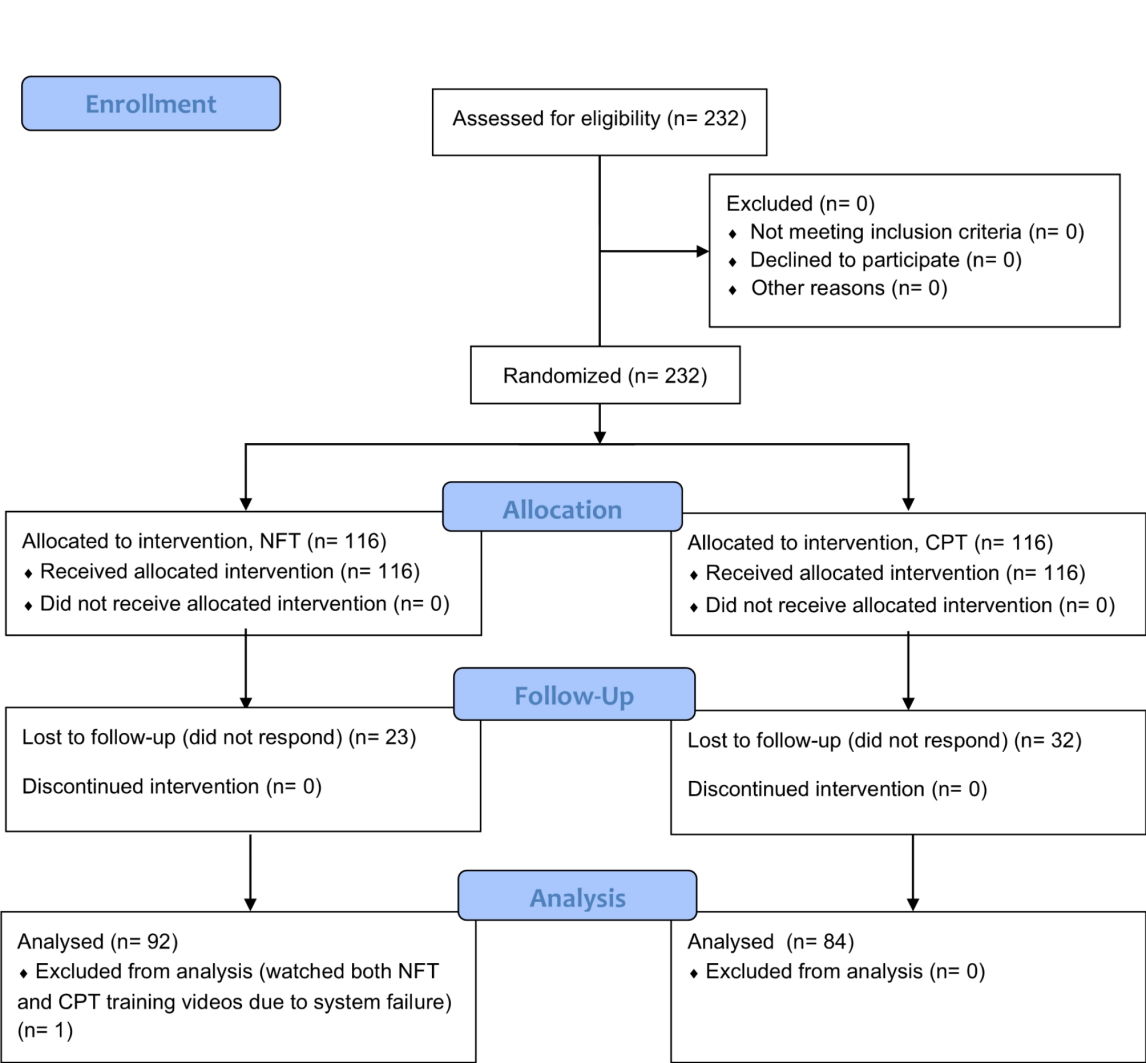
Diagram of the study and flow of participants

Figure 1 outlines the trial procedure. From September 12th, 2019, students’ mobile group chatrooms were used to distribute recruitment announcements with a link to the research sign-up page. A total of 232 out of the 260 eligible students who signed up for participation arrived at the computer labs where the training was to be held. Upon arrival at the lab, lab assistants provided students with a pencil and a paper pad and guided them to their individual computers. Then, they were instructed to direct their browsers to the study website housed on Qualtrics, a secure online survey system. The first page of the website provided information about the study and an informed consent form. Students who consented to participate were randomly assigned to either NF-TT (*n* = 116) or CP-FT (*n* = 116). Randomization was completed using Qualtrics’ randomizer function that uses simple randomization; thus, both participants and investigators were blinded to the intervention assignments.

Students in both training groups completed a baseline questionnaire, which included a task for estimating positive predictive value (PPV), a task for estimating negative predictive value (NPV), and a question about whether they had any class covering test accuracy indices. (The following subsection provides detailed examples of tasks, including solution processes.) Then, students watched a 15-minute-long training video clip respective to the assigned group. We created the training video clips based on previous research [29]. (Supplementary Appendix A provides a detailed description of the training videos and training video URLs.) After watching the video clips, students completed a post-intervention questionnaire including five PPV/NPV estimation tasks. During the training session, the lab assistants instructed students to work individually at their own pace and utilize the pencils and paper pads provided while refraining from using calculators. The restriction on calculator usage was imposed for two reasons: (1) to encourage participants to record their computations on the provided paper pads, enabling us to examine their approach to solving Bayesian reasoning tasks, and (2) to simulate typical clinical situations where medical professionals often interpret and explain test results without immediately resorting to calculators, thus improving the study’s ecological validity. Training sessions lasted around 50 min on average, including completing the baseline and post-intervention questionnaires. Students received 35,000 KRW, equivalent to around 27 USD, for participating in training sessions.

One month after the training session, we emailed students a follow-up questionnaire so they could complete it remotely at their convenience; we also sent a reminder of the questionnaire via text messaging. The follow-up questionnaire included five PPV/NPV estimation tasks and two conditional probabilistic reasoning tasks in non-medical contexts. (The following subsection provides a comprehensive description of the tasks.) After completing the follow-up questionnaire, we debriefed the students and provided them with 15,000 KRW, which is approximately equivalent to 12 USD.

### Outcome measures

The primary outcome of this study was the accuracy of PPV/NPV estimation, or the percentage of correct solutions to PPV/NPV estimation tasks. PPV/NPV estimation accuracy was assessed at three different time points: baseline, post-intervention (immediately after training), and follow-up (one month after training). The specific questions used at each time point varied but were the same for all participants at each time point. The questions were designed to be of similar difficulty across all time points. At baseline, participants were given two tasks: one PPV and one NPV estimation task. They were given five tasks at both post-intervention and follow-up: four PPV and one NPV estimation tasks. Two post-intervention PPV estimation tasks were repeatedly used at follow-up. We adapted four tasks from previous studies [6, 7, 35, 44] and developed the others to diversify estimation tasks regarding a target condition and a requested estimation (i.e., NPV or PPV). We deliberately chose different tasks for each time period rather than repeating the same tasks. We made this decision to reduce the impact of practice and make sure that any performance improvements are due to genuine learning and skill development rather than just becoming more familiar with specific items. By using different but conceptually similar tasks, we can more confidently evaluate participants’ ability to apply their knowledge to new situations, which is an important aspect of medical decision-making. This approach also keeps participants engaged and lowers the risk of them memorizing specific solutions to problems.

**Fig. 2:**
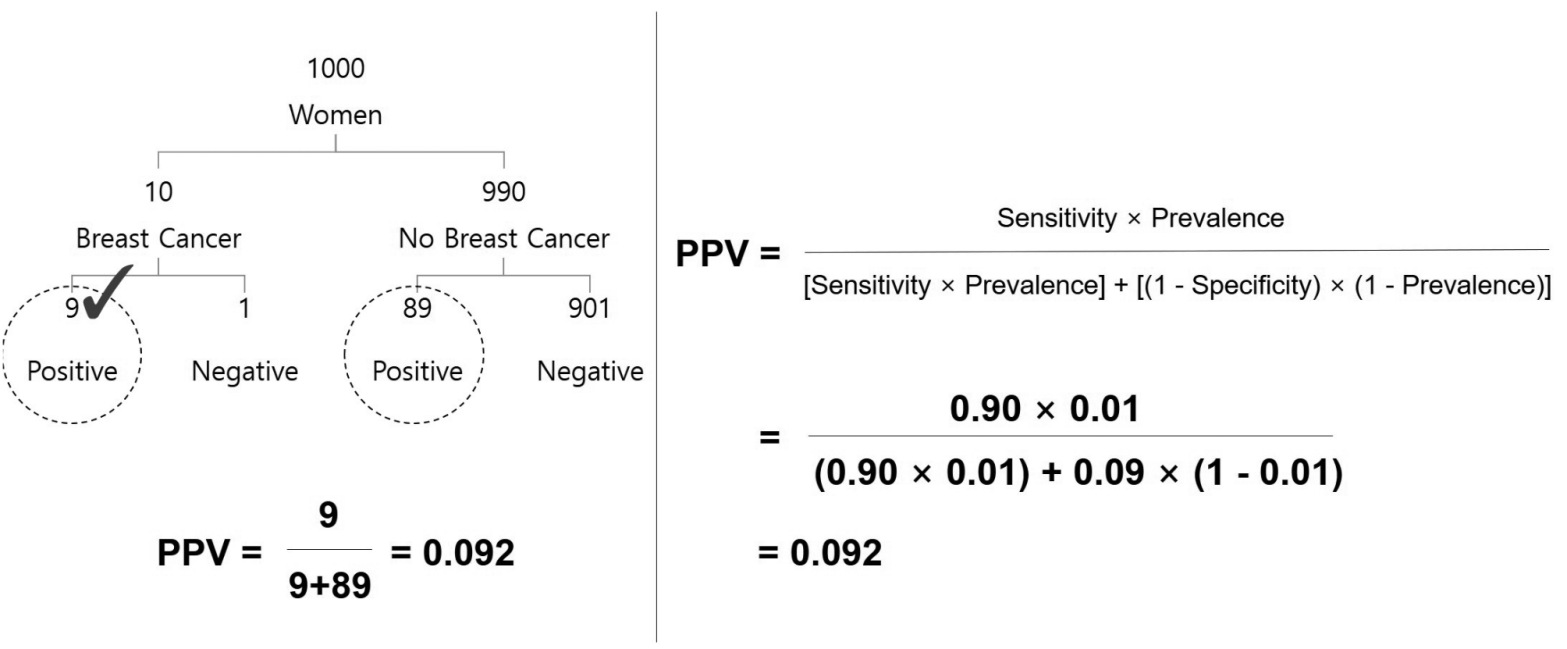
Solution process for calculating the PPV of mammography using a natural frequency tree (on the left) and conditional probability formula (on the right)

All the tasks were text-based and provided statistical information in probability format. For example, one baseline task asking about the PPV of mammography was adapted from Eddy [5] (i.e., “The prevalence of breast cancer in women in their 60s is 1%. If a woman has breast cancer, there is a 90% probability that she will get a positive mammogram. If a woman does not have breast cancer, there is a 9% probability that she will get a positive mammogram. Which of the following is the closest approximation of the probability that a woman who tests positive has breast cancer?”). Figure 2 illustrates the process for calculating the PPV of mammography using natural frequency tree and conditional probability formula. Although previous studies used fill-in-the-blank-type questions requesting respondents to record their estimations, there has been an ongoing debate regarding whether the facilitative effect of natural frequencies depends on loose or stringent criteria applied when coding submitted estimations [17, 45]. Thus, we used multiple-choice questions to minimize the subjectivity in classifying students’ estimations and the possibility of any systematic misclassification errors. Each estimation task had four answer choices, the order of which was randomized: one correct or closest answer and three distractors. The distractors of PPV estimation tasks were prevalence, sensitivity, and ‘sensitivity minus false-positive rate,’ all reported as the most common incorrect answers to PPV estimation tasks [14]. The distractors of NPV estimation tasks were formulated similarly. All the tasks were reviewed and revised with input from a practicing physician and a public health professional with a medical background. (See Table S1 for the statistics involved in all tasks and the correct Bayesian solutions).

The secondary outcome of interest was the transfer of learning, which refers to the ability to apply knowledge, skills, or modes of thought acquired in one learning context to a new context [46]. In this study, transfer of learning was operationalized as the percentage of correct solutions to two conditional probabilistic reasoning tasks in non-medical contexts adapted from a previous study [32]. One task involved detecting defective product materials (i.e., the probability that material that has been identified as defective came from a particular supplier), and the other involved detecting incorrect tax returns (i.e., the probability that a taxpayer’s return that has been identified as having an error has an error). We only assessed the transfer of learning at follow-up.

### Sample size, power calculation, and statistical analysis

As stated in the trial registry entry, we initially developed this trial to compare the effects of NF-TT and CP-FT and to examine the effect of time within each training group. Consistent with this aim, originally, we planned to analyze data using a mixed ANOVA with one between-subject factor (training group) and one *within-subject factor* (assessment time points). A priori sample size calculation was performed using G*power version 3.1.9.7 [47]. Since previous studies [2, 29, 30] did not have the same experimental design or apply the same statistical analysis strategy, we did not refer to their effect sizes. With a priori assumptions of effect size *f* = 0.45, *α* = 0.05, the power of the test (1–*β*) = 0.95, and a correlation among repeated measures: *r* = .90, a minimum total sample size of 60 was estimated (i.e., 30 per group). Allowing for an attrition rate of 50%, a total sample size of 120 participants was required for this trial (i.e., 60 per group). Over the course of the study design, we realized that students’ prior educational training would be an important source of heterogeneity in the effectiveness of NF-TT that needs to be examined. As a result, we increased the targeted sample size to 240 to prevent underpowered statistical analyses, and 232 students—116 in each training group—completed their training sessions. However, data cleaning revealed that network disruption exposed one student in NF-TT to a CP-FT video clip. As a result, we excluded the student from the analysis.

After changing the targeted sample size and statistical analysis plan following trial registration, we also conducted a post-hoc power calculation using G*power. With a sample size of 231 students who participated in the training session (115 in NF-TT; 116 in CP-FT) and a two-tailed alpha of 0.05, the power to detect differences between the two groups was 96% for a medium-sized effect (Cohen’s *d* = 0.50) and 32% for a small-sized effect (Cohen’s *d* = 0.20). The sample size of 176 who completed the follow-up test as well (92 in NF-TT; 84 in CP-FT) would provide 90% power to detect the medium size effect and 25% power to detect the small size effect.

The principle of intention to treat was applied to all analyses, preserving the original randomization. Of note, 76% of NF-TT participants and 78% of CP-FT participants used frequencies and probabilities, respectively, to calculate PPV/NPV, indicating that most of the participants generally *complied with* the allocated training regime (see Supplementary Appendix B and Table S2 for more information about the procedure to code the hand calculations students left on the provided notepads). We conducted Chi-square and t-tests to evaluate the balance between the two training groups concerning baseline variables. Since the outcome measures were not normally distributed, Wilcoxon signed ranks test and Mann-Whitney U tests were conducted for within-group and between-group comparisons respectively. As a post-hoc subgroup analysis, we also performed Mann-Whitney U tests. IBM SPSS Statistics 25 was used to perform the statistical analyses.

## Results

### Study participants

Of the 231 participants included in the analysis, 145 (63%) were male, and 208 (90%) were in their twenties. Participants were proportionately at different stages of their studies, equally representing all years in medical school. Approximately 60% of the participants had received prior educational training on the indices of test accuracy. The mean PPV/NPV estimation accuracy at baseline was 69% (55% of participants solved both baseline tasks correctly), which was surprisingly higher than expected based on the previous studies. Participants in the NF-TT and CP-FT groups were not statistically different in terms of gender, age, attending school, year of study, prior training, and the baseline PPV/NPV estimation accuracy at the 0.05 significance level. In addition, the proportion of participants who achieved 100% accuracy at baseline and the proportion of participants who completed the follow-up questionnaire were not statistically different between the groups at the 0.05 significance level (Table 1). The participants did not report any unexpected harm or negative events due to their participation.

**Table 1:** Characteristics of study participants (*N* = 231) by training group

		NF-TT (*N* = 115)	CP-FT (*N* = 116)	*p*-value
Gender	Male	70 (61%)	75 (65%)	0.55
	Female	45 (39%)	41 (35%)	
Age	–19	10 (9%)	12 (10%)	0.55
	20–29	105 (91%)	103 (89%)	
	30–39	0 (0%)	1 (1%)	
Attending school	School A	57 (50%)	59 (51%)	0.88
	School B	58 (50%)	57 (49%)	
Year of study	Year 1	19 (17%)	20 (17%)	0.82
	Year 2	16 (14%)	18 (16%)	
	Year 3	25 (22%)	18 (16%)	
	Year 4	17 (15%)	23 (20%)	
	Year 5	19 (17%)	19 (16%)	
	Year 6	19 (17%)	18 (16%)	
Prior training	Absent	47 (41%)	47 (41%)	0.96
	Present	68 (59%)	59 (59%)	
Baseline accuracy (%)		68.70	69.40	0.68
Participants with 100% baseline accuracy		60 (52%)	67 (58%)	0.43
Participants who completed follow-up		92 (80%)	84 (72%)	0.18

Note: percentages may not add up to 100% due to rounding

### Effectiveness of NF-TT versus CP-FT

#### Overall effectiveness

Table 2 presents the means and standard deviations of the accuracy of PPV/NPV estimation and transfer of learning across training groups, and the presence or absence of prior training. Participants in NF-TT and CP-FT were not statistically different in their PPV/NPV estimation accuracy at both post-intervention (*M*_NF−TT_=87.13%; *M*_CP−FT_*=*86.03%, *p =* .86, Cohen’s *d* = 0.02) and follow-up (*M*_NF−TT_=72.39%; *M*_CP−FT_*=*68.10%, *p =* .40, Cohen’s *d* = 0.10). Transfer of training did not occur differently between the two training groups either (*M*_NF−TT_=75.54%; *M*_CP−FT_*=*71.43%, *p =* .41, Cohen’s *d* = 0.11). Both NF-TT and CP-FT showed statistically significant within-group changes in the accuracy of PPV/NPV estimation over the study period. The estimation accuracy increased from baseline (*M*_NF−TT_=68.70%; *M*_CP−FT_=69.40%) to post-intervention (*M*_NF−TT_=87.13%, *Z*= − 5.10, *p* < .001; *M*_CP−FT_=86.03%, *Z*=–4.03, *p* < .001) and then decreased from post-intervention to follow-up (*M*_NF−TT_=72.39, *Z*=–4.19, *p* < .001; *M*_CP−FT_=68.10%, *Z*=–4.10, *p* < .001). Looking at the net effect over the entire study period, for NF-TT group, there was a small, non-significant improvement from baseline (*M* = 68.70%) to follow-up (*M* = 72.39%, *p* = .08). CP-FT group showed a slight, non-significant decline from baseline (*M* = 69.40%) to follow-up (*M* = 68.10%, *p* = .80).

**Table 2:** Means (SDs) of the accuracy of PPV/NPV estimation and transfer of learning as function of training group and prior training

	Intervention group	PPV/NPV estimation (%)				Transfer of learning (%)
		Baseline	Post	Follow-up		Follow-up
Overall (*N* = 231)	NF-TT (*n* = 115)	68.70 (36.55)	87.13 (19.86)	72.39 (29.63)		75.54 (38.15)
	CP-FT (*n* = 116)	69.40 (39.44)	86.03 (21.30)	68.10 (33.17)		71.43 (39.03)
Prior training (*n* = 137)	NF-TT (*n* = 68)	68.38 (35.52)	87.35 (21.27)	70.88 (28.55)		71.05 (40.03)
	CP-FT (*n* = 69)	69.57 (38.55)	85.22 (22.14)	73.58 (33.00)		74.53 (38.76)
No prior training (*n* = 94)	NF-TT (*n* = 47)	69.15 (38.38)	86.81 (17.83)	74.86^*^ (31.56)		82.86^*^ (34.18)
	CP-FT (*n* = 47)	69.15 (41.12)	87.23 (20.18)	58.71^*^ (31.81)		66.13^*^ (39.55)

^*^Difference is significant at the 0.05 level

#### Impact of prior training on training effectiveness

Participants with prior training exhibited a pattern of results consistent with that obtained for the entire sample. We did not observe any significant group difference between CP-FT and NF-TT in terms of the accuracy of PPV/NPV estimation at post-intervention (*p* = .51) and at follow-up (*p* = .32) and transfer of learning (*p* = .62). However, participants without prior training exhibited a distinct pattern of results. Specifically, at follow-up, NF-TT participants achieved a higher PPV/NPV estimation accuracy than CP-FT participants (*M*_NF−TT_=74.86; *M*_CP−FT_*=*58.71%, *p =* .02, Cohen’s *d* = 0.58). Transfer of learning also appeared to occur significantly more among NF-TT participants than CP-FT participants (*M*_NF−TT_=82.86%; *M*_CP−FT_*=*66.13%, *p =* .04, Cohen’s *d* = 0.43).

**Fig. 3:**
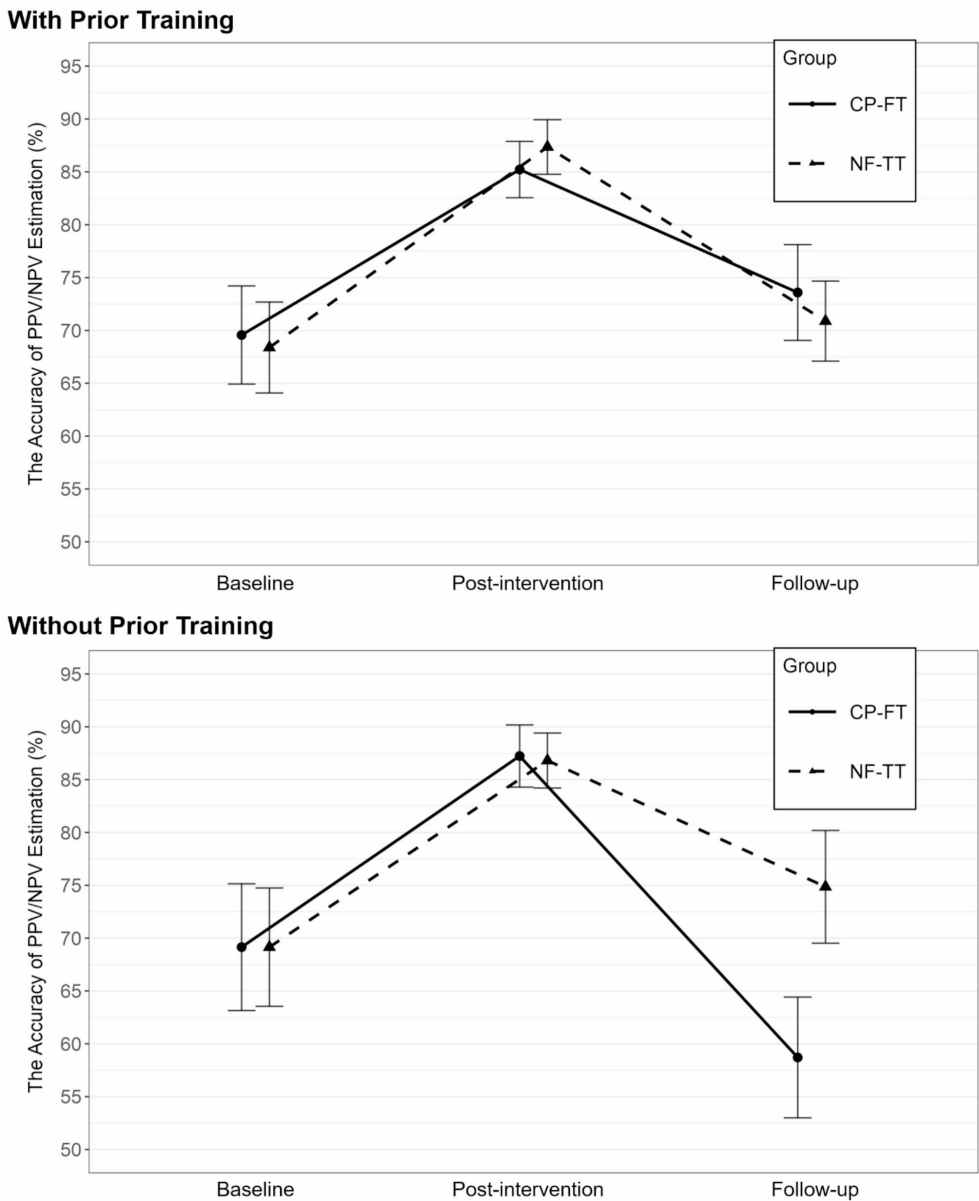
Changes in the accuracy of PPV/NPV estimation across time points by prior training Note: Error bars represent standard errors of the mean

In terms of overall change from baseline to follow-up among participants with prior training, neither NF-TT nor CP-FT training showed a statistically significant improvement (NF-TT: *M*_baseline_ =68.38%, *M*_follow−up_=70.88, *p* = .45; CP-FT: *M*_baseline_ =69.57%, *M*_follow−up_=73.58%, *p* = .35). Similarly, no statistically significant change was shown from baseline to follow-up in either training group among participants without prior training (NF-TT: *M*_baseline_ =69.15%, *M*_follow−up_=74.86%, *p* = .10; CP-FT: *M*_baseline_ =69.15%, *M*_follow−up_=58.71%, *p* = .50). Figure 3 shows a visual representation of changes in the accuracy of PPV/NPV estimation across time points by prior training.

#### Post-hoc subgroup analysis: training effectiveness based on prior training and baseline performance

As a post-hoc analysis, we examined the effects of NF-TT vs. CP-FT at both post-intervention and follow-up, considering prior training and baseline accuracy. This analysis was motivated by the observation that more than half of the participants achieved 100% baseline PPV/NPV estimation accuracy, potentially influencing training effectiveness. Due to space constraints, we report only interesting patterns here; full results are in Table S3 of the supplementary materials. At post-intervention, no significant differences were found between training groups across all subgroups. However, at follow-up, for participants who achieved 100% baseline accuracy, the effectiveness of the training methods differed based on prior training. Among those with prior training, CP-FT appeared more effective at follow-up (*M*_CP−FT_*=*84.83%, *M*_NF−TT_=77.14%, *p =* .04, Cohen’s *d* = 0.51). However, for those without prior training, NF-TT was more effective (*M*_NF−TT_=78.75%, *M*_CP−FT_*=*60.00%, *p =* .02, Cohen’s *d* = 0.87). For participants with less than 100% baseline accuracy, no significant differences were found between training groups in estimation accuracy at both post-intervention and follow-up, regardless of prior training. Similarly, transfer of learning performance showed no significant differences between groups across all subgroups.

## Discussion

We evaluated the effectiveness of natural frequency tree-based training (NF-TT) in improving South Korean medical students’ ability to estimate predictive values of screening tests compared to conventional conditional probability formula-based training (CP-FT). Both NF-TT and CP-FT significantly improved the medical students’ PPV/NPV estimation accuracy at post-intervention. Across all the students, NF-TT and CP-FT were not significantly different in their effectiveness in improving PPV/NPV estimation and transfer of learning. However, for students without prior training, NF-TT was more effective than CP-FT. This indicates that students who have never taken a class covering test accuracy indices and predictive value are more likely to remember and better apply the training instruction at follow-up when they receive NF-TT rather than CP-FT.

Our research contributes to the existing literature by examining NF-TT in the context of South Korean medical education, a setting with unique characteristics. Although previous studies with similar methodologies typically showed the superior effectiveness of NF-TT [2, 29, 30], our findings are inconsistent with those results. The extreme competitiveness of South Korean medical schools [48] may result in a student population with exceptionally high preexisting knowledge and skills relevant to conditional probabilistic reasoning. This high baseline capability could have reduced the differential impact of NF-TT and CP-FT, as students may already have strong foundational skills regardless of the training method. The quality of CP-FT implemented in this study may also explain the unexpected finding. To ensure a fair comparison, we intended for both NF-TT and CP-FT to provide as equivalent information as possible. Thus, like the NF-TT video clip, the CP-FT video clip explained how test accuracy indices are derived and what each component of a conditional probability formula means using a 2 × 2 frequency table. It then demonstrated how to solve PPV/NPV estimation tasks using the formula. This is consistent with South Korea’s typical approach to teaching diagnostic accuracy measures. This approach likely enabled CP-FT participants to understand the reasoning behind the formula rather than simply memorizing it. Notably, previous research has shown that training using 2 × 2 frequency tables can be as effective as frequency trees in improving Bayesian reasoning [49]. In our study, the shared foundational knowledge of frequency-based representations of diagnostic accuracy using the 2 × 2 table in both training groups may explain why the training group difference was less pronounced than in previous studies, where participants might not have had this baseline understanding.

There was also an unexpected finding about the effectiveness of CP-FT. While CP-FT was significantly less effective for students without prior training, it was more effective for students who were also already proficient in PPV/NPV estimation with prior training. There are two possible explanations for this. First, CP-FT would have provided opportunities for pre-trained students already proficient in PPV/NPV estimation to review what they already knew. Second, students with prior training might have been inclined to stick to the previously learned formula-based method rather than to adopt a newer or more straightforward natural frequency tree-based approach to solve PPV/NPV estimation tasks [50], which is a good exemplar of the tendency known as the Einstellung effect [51]. In fact, NF-TT participants with prior training utilized the exact strategy they had been taught (i.e., the natural-frequency tree diagrams) to a lesser extent than those without prior training (28% vs. 50%) (see Table S2 for more information).

This study makes several insightful contributions. First, this study informs medical education that introducing NF-TT early in the medical school curricula, specifically before students are exposed to a pervasive conditional probability formula-based approach to PPV/NPV estimation, would be most effective, as it demonstrates that students without relevant prior training benefit more from NF-TT. Given that learning from NF-TT was relatively more stable and transferable than learning from CP-FT (see the results of the overall analysis in Table 2), teaching medical students how to apply a natural frequency tree-based approach to Bayesian reasoning in the early stages of their educational program may have the potential to benefit them more perpetually until later in their academic and professional careers. Second, while CP-FT was ineffective for students without prior training, it was more effective for students with relevant training and 100% accuracy in PPV/NPV estimation at baseline. One major lesson from these findings is that NF-TT may not equally empower medical students and professionals to interpret test results accurately.

The PPV/NPV estimation accuracy increased significantly from baseline to post-intervention but then decreased at the follow-up, nearly returning to baseline levels. Both the one-month gap and the change in data collection methods, from in-lab assessments (baseline/post-intervention) to remote assessments (follow-up), could account for this lack of sustained improvement. This methodological change represents both a limitation and a strength of our study. The remote follow-up may have affected participants’ engagement or effort levels due to the absence of direct supervision and the presence of real-world distractions, potentially influencing their performance and making it difficult to isolate the true effect of the one-month gap from the observed performance declines. However, the methodological change also enhances the ecological validity of our findings as medical students and professionals often need to apply their skills in unsupervised, real-world settings. Thus, follow-up results may more accurately reflect how these skills persist and are applied in practical contexts. Despite the potential influence of the methodological change, our results provide valuable insights into how skills obtained through formal training persist over time. As discussed in detail earlier, we found that the effectiveness of the training methods differed based on participants’ prior training. While we cannot ascertain the effectiveness beyond our one-month follow-up period, these results highlight the complex nature of skill acquisition and retention in Bayesian reasoning. They underscore the need for further research into how different training methods impact skill maintenance in various subgroups of learners.

This study has some limitations. First, our NF-TT combined two elements, natural frequencies and tree diagrams, that have shown promise in improving Bayesian reasoning. We acknowledge that this design does not allow us to disentangle the individual effects of natural frequencies and tree diagrams. As a result, we cannot definitively attribute observed differences to either element alone. However, the direct comparison between NF-TT and CP-FT offers valuable insights for evaluating alternatives to current educational practices in South Korean medical schools. It assesses the combined effect of these elements in a real-world context relevant to potential curriculum changes. Future studies could aim to disentangle these effects. Second, our decision to restrict calculator use in the training session could have disproportionately penalized the CP-FT group. Future studies might allow calculator use or compare performance with and without calculators to isolate the effects of the different training methods better. Third, although our one-month follow-up provides initial insights, it is insufficient for evaluating the long-term effectiveness of NF-TT and CP-FT. Longitudinal designs that track students’ Bayesian reasoning abilities over extended periods (e.g., 6 months, a year, or longer) would enhance future research. Such efforts would more accurately assess the sustained improvement in Bayesian reasoning and provide crucial insights for incorporating these training methods into medical education. Fourth, this study did not consider the effects of internal and external motivation. Participants may have had enough internal motivation to solve the academic-style probabilistic reasoning tasks as they were presented like exams. We find this argument quite reasonable because the computer labs where the training sessions were held are typically used as Computer-Based Testing rooms. Furthermore, the financial incentives for participation incentives might have motivated students to perform well in this study [16, 52]. The study’s unusually high accuracy of PPV/NPV estimation at baseline might be due to the internal and external motivation that students received during the participation process [39, 44], and therefore, these results may not apply to other students lacking similar motivation. There are other limitations related to the assessment methods. Firstly, since we only used multiple-choice questions, some correct answers may have resulted from guessing rather than genuine understanding. However, in our analysis of participants’ handwritten calculations, we did not find instances of pure guesswork. Moreover, the high accuracy rates observed (over 80% post-intervention and around 70% at follow-up) are higher than the 25% expected from random guessing on our four-option multiple-choice questions, indicating that participants were not simply guessing. In addition, the joint probability, which is also often mistaken for conditional probabilities in Bayesian reasoning tasks [53], was not included as a distractor in our multiple-choice questions. Including joint probability as a response option may have yielded further insights into the various sorts of mistakes participants make in their reasoning. Secondly, we only assessed the transfer of learning at follow-up, missing the chance to monitor the development of Bayesian reasoning skills over time. To address these limitations, future studies may use a mix of multiple-choice and open-ended questions, incorporate a more comprehensive set of distractors (including joint probabilities), and assess transfer at multiple time points. Last, the limitations of subgroup analyses (e.g., false negatives due to inadequate statistical power) should also be considered when interpreting this study’s findings [54]. 

Even though some points of concern exist, this study evaluated the effectiveness of NF-TT using a rigorous research design, which was crucial for establishing a foundation for, or reconsidering, the adoption of NF-TT. This study also advances the field’s limited understanding of factors that influence the effectiveness of NF-TT by examining how the facilitative effect of NF-TT differed across different subsets of students with different educational experiences in medical schools and varying degrees of baseline proficiency in PPV/NPV estimation. We expect that future research will complement and expand our study by investigating other contextual or student-specific factors that may contribute to variances in NF-TT effectiveness. It is also possible that NF-TT could be a practical and useful way to support health professionals in helping patients—who are probably ignorant about the predictive values of medical tests—discuss conditional probabilistic reasoning, given its superior effectiveness in the absence of prior training. In that sense, NF-TT could be a simple but important action step to move closer to the ideal of universal design that supports informed decision-making about test options.

## Electronic supplementary material

Below is the link to the electronic supplementary material.

Supplementary Material 1

## Data Availability

Consent for data sharing was not obtained but the presented data are anonymized, and the risk of identification is low. The datasets generated and/or analyzed during the current study are not publicly available due to ethical restrictions but are available from the corresponding authors upon reasonable request.

## Abbreviations

CP-FT: Conditional probability formula-based training
NF-TT: Natural frequency tree-based training
NPV: Negative predictive value
PPV: Positive predictive value
