# Supplementary material for: Natural frequency tree- versus conditional probability formula-based training for medical students’ estimation of screening test predictive values: a randomized controlled trial
Source: BMC Med Educ. 2024 Oct 24;24:1207. doi: 10.1186/s12909-024-06209-0 (PMC11515371; doi:10.1186/s12909-024-06209-0)
Supplement: Supplementary file 1 — Supplementary Material 1 [file 12909_2024_6209_MOESM1_ESM.docx]

**Appendix A. Training Videos**

Both NF-TT and CP-FT videos started by explaining the indices of test accuracy (e.g., sensitivity and specificity) and how they are derived using a 2×2 frequency table (disease absent/present × test positive/negative). This introduction was essential to lay the groundwork for understanding the basic concepts and how these concepts interact with real-world factors (i.e., disease prevalence) to influence the interpretation of test results. In NF-TT, the video then showed how to solve the two baseline PPV/NPV estimation tasks by converting the test accuracy indices presented in probabilities into natural frequencies and organizing those natural frequencies into a tree diagram. The translation process involved four steps: (a) selecting a population (such as 1,000 women) and calculating the number of individuals in the population with the disease based on the disease prevalence (e.g., if the prevalence is 1%, 10 women have the disease); (b) utilizing the test's sensitivity to determine the number of individuals who both have the disease and test positive (e.g., if the sensitivity is 90%, 9 out of 10 women with the disease test positive); (c) utilizing the test's false positive rate to determine the number of individuals who do not have the disease but still test positive (e.g., if the false positive rate is 9%, 89 of 990 women without the disease test positive); (d) comparing the number obtained in Step 2 with the sum of those obtained in Steps 2 and 3 to determine the probability that a person who tests positive actually has the disease $(9/(9+89)*100=0.0918\approx9\%)$. The content and sequence of the CP-FT video were precisely the same as those of the NF-TT video. The only difference was that CP-FT video extensively explained a conditional probability formula and its components and demonstrated how to plug the test accuracy indices presented in probabilities into the formula to solve PPV/NPV estimation tasks (NF-TT video: <https://youtu.be/maXMPAbh9nQ>; CP-FT video: <https://youtu.be/AeYxsdygHCM>).

**Appendix B: Coding Bayesian reasoning strategy and approach students used**

On the provided notepads, two hundred twenty-one students recorded their calculations and notes outlining how they arrived at their solutions. Two writers (SK and SK) looked at a random selection of the notepads, came up with a list of codes, and combined those codes to create a coding scheme inductively. The generated codes are as follows: (a) tree diagram with natural frequencies; (b) tree diagram with probabilities; (c) conditional probability formula; (d) 2×2 table with natural frequencies; (e) 2×2 table with probabilities; and (f) the unidentifiable. Then, the two authors independently coded 25% of all notes according to the coding scheme. Multiple coding was allowed when more than one coding category was applicable. Since inter-coder reliabilities measured with Cohen’s kappa ranged from. 85 to 1.00, the remaining notes were coded by one author. The researchers in the study coded the notes taken by the students into two variables—estimation *approach* and estimation *strategy*. Firstly, the participants who used a tree diagram or a 2x2 table with natural frequencies were coded as having applied the frequency-based approach to Bayesian reasoning, as they were trained. Then, the participants who used a conditional probability formula, tree diagram or 2x2 table with probabilities were coded as having applied the probability-based approach to Bayesian reasoning, as they were trained. Secondly, the researchers used a stricter criterion to code the participants who applied the exact estimation strategy as trained. Only the participants who used tree diagrams with natural frequencies from the NF-TT group and the participants who used the conditional probability formula from the CP-FT group were coded as having applied the exact estimation strategy as trained.

**Table S1.** Summary of PPV/NPV estimation tasks with statistical information provided, the correct Bayesian solutions, and percentages (SDs) of correct answers by training group

|  | Assessment time point | | | Prevalence | Sensitivity | Specificity | False-positive rate | Correct Bayesian solution | | Percentage (SD) of correct answer by training group | |
| --- | --- | --- | --- | --- | --- | --- | --- | --- | --- | --- | --- |
|  | Baseline | Post | Follow-  up |  |  |  |  | PPV | NPV | NF-TT (n=115) | CP-FT (n=116) |
| Mammography(1) | ○ |  |  | 1% | .90% |  | 9% | 9.2% |  | 60.00 (49.20) | 62.07 (48.73) |
| Test for Disease X | ○ |  |  | 10% | 75% | 96% |  |  | 97% | 77.39 (42.01) | 76.72 (42.44) |
| Test for MERS-CoV RNA |  | ○ |  | 3% | 93.9% |  | 0.4% | 87.9% |  | 82.61 (38.07) | 85.34 (35.52) |
| Genetic test for type 2 Diabetes(2) |  | ○ |  | 0.4% | 50% |  | 50% | 0.4% |  | 93.04 (25.55) | 93.10 (25.45) |
| HL-Antigen-B27 test for ankylosing spondylitis(3) |  | ○ |  | 5% | 92% |  | 8% |  | 99.5% | 85.22 (35.65) | 84.48 (36.36) |
| Prostate-Specific Antigen test for prostate cancer |  | ○ | ○ | 7.7% | 21% | 91% |  | 16.4% |  | Post:  83.48 (37.30)/  Follow-up:  58.26 (49.53) | Post:  80.17 (40.04)/  Follow-up:  47.41 (50.15) |
| Ultrasound screening for thyroid cancer |  | ○ | ○ | 7% | 94% | 66% |  | 17.2% |  | Post:  91.30 (28.30)/  Follow-up: 60.00 (49.20) | Post:  87.07 (33.70)/  Follow-up: 54.31 (50.03) |
| A fictitious test for a fictitious disease |  |  | ○ | 60% | 95% |  | 75% |  | 76.9% | 60.87 (49.02) | 53.45 (50.10) |
| ELISA for HIV(4) |  |  | ○ | 0.01% | 99.8% |  | 0.01% | 50% |  | 46.09 (50.07) | 44.83 (49.95) |
| Genetic test for breast cancer(2) |  |  | ○ | 8% | 5% |  | 0.29% | 60% |  | 64.35 (48.11)* | 46.55 (50.10)* |

*Note*. Tasks without citation of reference are developed for this study

**Table S2.** Mean application rates (0-1 scale) of learned approaches and strategies by training group

|  |  | Application of learned approach^1^ | Application of learned strategy^2^ |
| --- | --- | --- | --- |
|  |  |  |  |
| *Overall* (n=231) |  |  |  |
|  | NF-TT (n=115) | .76  (.43) | .37^***^  (.48) |
|  | CP-FT (n=116) | .78  (.41) | .79^***^  (.41) |
| *Subgroup by prior training* |  |  |  |
| Prior training (n=137) | NF-TT (n=68) | .80  (.40) | .28^***^  (.45) |
|  | CP-FT (n=69) | .80  (.41) | .81^***^  (.40) |
| No prior training (n=94) | NF-TT (n=47) | .70  (.47) | .50^**^  (.51) |
|  | CP-FT (n=47) | .76  (.43) | .76^**^  (.43) |
| *Subgroup by baseline accuracy* |  |  |  |
| Baseline accuracy = 100% (n=127) | NF-TT (n=60) | .76  (.43) | .34***  (.48) |
|  | CP-FT (n=67) | .77  (.43) | .76***  (.43) |
| Baseline accuracy < 100% (n=104) | NF-TT (n=55) | .75  (.43) | .40***  (.49) |
|  | CP-FT (n=49) | .80  (.40) | .83***  (.38) |

*Note*. Values represent mean rates on a scale from 0 to 1, where 1 indicates 100% application.

^1^ When compliance to training was less strictly conceptualized as whether students used the information format they had been trained in (i.e., frequencies or probabilities) (0=No, 1=Yes)

^2^ When compliance to training was more strictly conceptualized as whether students applied the exact strategy for conditional probability reasoning they had learned through training (i.e., natural frequency tree or conditional probability formula) (0=No, 1=Yes)

^**^Difference is significant at the .01 level; ^***^Difference is significant at the .001 level

**Table S3.** Means (SDs) of the accuracy of PPV/NPV estimation and transfer of learning as a function of training group, prior training, and baseline estimation accuracy

|  |  | PPV/NPV estimation (%) | | |  | Transfer of learning (%) |
| --- | --- | --- | --- | --- | --- | --- |
|  |  | Baseline | Post | Follow-up |  | Follow-up |
| Baseline accuracy = 100% (n=127) | NF-TT (n=60) | 100.00  (0.00) | 91.66  (14.40) | 77.73  (25.60) |  | 82.95  (32.25) |
|  | CP-FT (n=67) | 100.00  (0.00) | 88.06  (19.40) | 78.89  (34.52) |  | 78.89  (34.52) |
| Prior training (n=73) | NF-TT (n=34) | 100.00  (0.00) | 92.35  (14.78) | 77.14^*^  (21.58) |  | 82.14  (31.07) |
|  | CP-FT (n=39) | 100.00  (0.00) | 90.26  (18.28) | 84.83^*^  (26.54) |  | 82.76  (33.48) |
| No prior training (n=54) | NF-TT (n=26) | 100.00  (0.00) | 90.77  (14.12) | 78.75^*^  (32.22) |  | 84.38  (35.21) |
|  | CP-FT (n=28) | 100.00  (0.00) | 85.00  (20.82) | 60.00^*^  (26.33) |  | 71.88  (36.37 |
| Baseline accuracy < 100% (n=104) | NF-TT (n=55) | 34.55  (23.32) | 82.18  (23.62) | 67.50  (32.39) |  | 68.75  (42.05) |
|  | CP-FT (n=49) | 27.55  (25.11) | 83.27  (23.58) | 58.97  (35.82) |  | 62.82  (42.49) |
| Prior training (n=64) | NF-TT (n=34) | 36.76  (22.39) | 82.35  (25.47) | 64.83  (33.23) |  | 60.34  (45.08) |
|  | CP-FT (n=30) | 30.00  (24.91) | 78.67  (25.15) | 60.00  (35.39) |  | 64.58  (42.93) |
| No prior training (n=40) | NF-TT (n=21) | 30.95  (24.88) | 81.90  (20.89) | 71.58  (31.49) |  | 81.58  (34.20) |
|  | CP-FT (n=19) | 23.68  (25.65) | 90.53  (19.29) | 57.33  (37.70) |  | 60.00  (43.09) |

^*^Difference is significant at the .05 level.

References for Supplemental Materials

1. Gigerenzer G, Hoffrage U. How to improve Bayesian reasoning without instruction: frequency formats. Psychol Rev. 1995;102(4):684–704.

2. Kurzenhäuser S. Natural frequencies in medical risk communication: Applications of a simple mental tool to improve statistical thinking in physicians and patients [doctoral dissertation]: Freie Universität Berlin; 2003.

3. Hoffrage U, Gigerenzer G. How to improve the diagnostic inferences of medical experts. Experts in science and society. Boston, MA: Springer; 2004. p. 249-68.

4. Gigerenzer G, Hoffrage U, Ebert A. AIDS counselling for low-risk clients. AIDS care. 1998;10(2):197-211.
